# Derived Categories of Hyper-Kähler Manifolds via the LLV Algebra

**DOI:** 10.1007/s00032-022-00358-x

**Published:** 2022-06-21

**Authors:** T. Beckmann

**Affiliations:** Max-Planck-Institute, Bonn, Viviatsgasse 7, 53111 Bonn, Germany

## Abstract

We mostly review work of Taelman (Derived equivalences of hyperkähler varieties, 2019, arXiv:1906.08081) on derived categories of hyper-Kähler manifolds. We study the LLV algebra using polyvector fields to prove that it is a derived invariant. Applications to the action of derived equivalences on cohomology and to the study of their Hodge structures are given.

## Introduction

In this note we discuss the *(bounded) derived category* $\mathrm {D}^b(X):=\mathrm {D}^b(\mathrm {Coh}(X))$ and its group of auto-equivalences $\mathrm {Aut}(\mathrm {D}^b(X))$ for projective hyper-Kähler manifolds *X*. The situation in dimension two, that is for K3 surfaces, is fairly well understood and we refer to [7, Sec. 10] for an overview. Therefore, we will only concentrate on the higher-dimensional case. More precisely, we mainly present the first part of Taelman’s paper [12].

These notes are, for the most part, light on derived categories and focus more on a different perspective of the Looijenga–Lunts–Verbitsky (LLV) Lie algebra ${\mathfrak {g}}(X)$ [8, 13] which will allow us to show the following.

### Theorem 1.1

(Taelman) A derived equivalence $\Phi :\mathrm {D}^b(X) \xrightarrow {\sim } \mathrm {D}^b(Y)$ between projective hyper-Kähler manifolds induces naturally a Lie algebra isomorphism

$$\begin{aligned} \Phi ^{{\mathfrak {g}}} :{\mathfrak {g}}(X) \xrightarrow {\sim } {\mathfrak {g}}(Y). \end{aligned}$$

The induced isomorphism of quadratic spaces

$$\begin{aligned} \Phi ^{\mathrm {H}}:\mathrm {H}^*(X,{\mathbb {Q}})\xrightarrow {\sim } \mathrm {H}^*(Y,{\mathbb {Q}}) \end{aligned}$$

is equivariant with respect to $\Phi ^{{\mathfrak {g}}}$.

The theorem will be proven in Sect. 5.

We start these notes by introducing the main objects of study and a collection of known results prior to [12]. Afterwards, we introduce a new Lie subalgebra of the (ungraded) endomorphism algebra $End (\mathrm {H}^*(X,{\mathbb {C}}))$ which is better suited for the study of derived categories. In the subsequent section we establish Theorem 1.1 via proving that the newly defined Lie subalgebra coincides with the well-known LLV Lie algebra ${\mathfrak {g}}(X) \otimes _{\mathbb {Q}} {\mathbb {C}}$ with scalars extended to the complex numbers. The next three sections will draw consequences from this result for the action of derived equivalences on cohomology and for Hodge structures of derived equivalent hyper-Kähler manifolds.

### Notation

We work over the complex numbers. Throughout these notes *X* and *Y* will be projective hyper-Kähler manifolds of dimension 2*n*. All functors will be implicitly derived.

## Derived Categories

### General Theory

For a thorough introduction to derived categories we recommend [7]. Let us recall one of the most important results in the study of derived equivalences proved by Orlov [9].

#### Theorem 2.1

Let *Z* and *T* be smooth projective varieties and $\Phi :\mathrm {D}^b(Z) \xrightarrow {\sim } \mathrm {D}^b(T)$ be an exact derived equivalence. Then $\Phi$ is isomorphic to a Fourier–Mukai functor, i.e. there exists ${\mathcal {E}}\in \mathrm {D}^b(Z\times T)$ such that

$$\begin{aligned} \Phi \simeq \mathrm {FM}_{{\mathcal {E}}}:={p_T}_*\circ ({\mathcal {E}} \otimes \_) \circ p_Z^{*}. \end{aligned}$$

Orlov’s result is in fact stronger in that it applies also to fully faithful exact functors between the derived categories of smooth projective varieties. The resulting isomorphism is an isomorphism of exact functors.

Moreover, a derived equivalence as in the theorem naturally induces isomorphisms of several invariants associated with the varieties such as (topological) *K*-theory [7, Sec. 5.2]. For us the most important invariant will be singular cohomology. Namely, every derived equivalence $\mathrm {FM}_{{\mathcal {E}}}$ induces a *cohomological Fourier–Mukai transform* $\mathrm {FM}^{\mathrm {H}}_{{\mathcal {E}}}$ given by the correspondence $v({\mathcal {E}})\in \mathrm {H}^*(Z\times T)$ where $v=\mathrm {ch(\_)}\sqrt{\mathrm {td}}$ is the Mukai vector. These are compatible via the Mukai vector, i.e. the following diagram commutes 
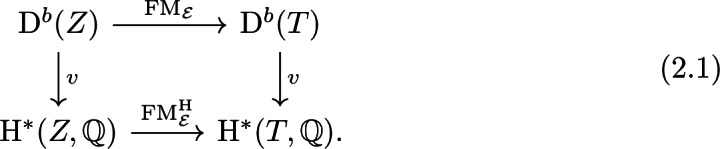
 Hence, the study of derived categories leads naturally to cycles on hyper-Kähler manifolds.

#### Remark 2.2

Let us mention properties of the cohomological Fourier–Mukai transform $\mathrm {FM}^\mathrm {H}_{{\mathcal {E}}}$.

- Since $v({\mathcal {E}})\in \oplus _p \mathrm {H}^{p,p}(Z\times T)$ is algebraic, the isomorphism $\mathrm {FM}^\mathrm {H}_{{\mathcal {E}}}$ respects the weight-zero Hodge structure on $\mathrm {H}^*(Z)$ (respectively $\mathrm {H}^*(T)$) given by
  $$\begin{aligned} \mathrm {H}^{-i,i}(Z)=\bigoplus _{q-p=i} \mathrm {H}^{p,q}(Z) \end{aligned}$$
  for $i\in {\mathbb {Z}}$ where the Hodge structure on the right-hand side is the usual one [7, Prop. 5.39].
- The isomorphism $\mathrm {FM}^\mathrm {H}_{{\mathcal {E}}}$ respects the generalized Mukai pairing, see [3].
- The cohomological Fourier–Mukai transform $\mathrm {FM}^\mathrm {H}_{{\mathcal {E}}}$ respects neither the cup product structure on cohomology nor the cohomological grading as can be seen by considering the equivalence given by tensoring with a non-trivial line bundle.

### Case of Hyper-Kähler Manifolds

We know that if a smooth projective variety *Z* is derived equivalent to a hyper-Kähler manifold *X*, then the dimensions of *X* and *Z* coincide and the canonical bundle $\omega _Z$ is trivial [7, Sec. 4]. Huybrechts and Nieper–Wißkirchen [5] have proven that *Z* must in fact also be an irreducible hyper-Kähler manifold.

## Recollection of the LLV Lie Algebra

We quickly recall the definition of the LLV Lie algebra introduced independently by Looijenga–Lunts [8] and [13]. For a more thorough discussion we refer to [2].

Let *X* be a hyper-Kähler manifold and $\lambda \in \mathrm {H}^2(X,{\mathbb {Q}})$ be a cohomology class. We attach to it the operator

$$\begin{aligned} e_\lambda :=\lambda \cup \_ \in End (\mathrm {H}^*(X,{\mathbb {Q}})) \end{aligned}$$

given by cup product with the class $\lambda$. We say that $\lambda$ has the *Hard Lefschetz property*, if for all *i* the mapsare isomorphisms. The class $\lambda$ is often called a *Hard Lefschetz class*. We denote by $h\in End (\mathrm {H}^*(X,{\mathbb {Q}}))$ the grading operator acting on $\mathrm {H}^{i}(X,{\mathbb {Q}})$ via $(i-2n) \mathrm {id}$. For a Hard Lefschetz class $\lambda \in \mathrm {H}^2(X,{\mathbb {Q}})$, the triple

$$\begin{aligned} (e_\lambda , h, f_\lambda ), \end{aligned}$$

where $f_\lambda$ is the dual Lefschetz operator, spans a Lie subalgebra of $End (\mathrm {H}^*(X,{\mathbb {Q}}))$ isomorphic to the Lie algebra $\mathfrak {sl}_2$.

### Definition 3.1

The *LLV Lie algebra* ${\mathfrak {g}}(X)$ is the Lie subalgebra of $End (\mathrm {H}^*(X,{\mathbb {Q}}))$ generated by all $\mathfrak {sl}_2$-triples $(e_\lambda , h, f_\lambda )$ for $\lambda \in \mathrm {H}^2(X,{\mathbb {Q}})$ Hard Lefschetz.

As said in the beginning, we refer to [2] or [8, 13] for more details and properties of ${\mathfrak {g}}(X)$. Our main goal is to relate the Lie algebra ${\mathfrak {g}}(X)$ to $\mathrm {D}^b(X)$. Note that since a cohomological Fourier–Mukai functor does not respect cup product nor grading, which are the defining properties of the LLV algebra, it is a priori not clear how this can be done. The main ingredient for it is the ring of polyvector fields, to be introduced now.

## Polyvector Fields

### Definition 4.1

The *ring of polyvector fields* $\mathrm {HT}^*(X)$ is the graded ${\mathbb {C}}$-algebra whose degree *k* part is

$$\begin{aligned} \mathrm {HT}^k(X):=\oplus _{p+q=k} \mathrm {H}^q\left( X,\bigwedge ^p {\mathcal {T}}_X\right) . \end{aligned}$$

The ring structure is induced from the exterior algebra.

For *X* a hyper-Kähler manifold we can choose a symplectic form $\sigma \in \mathrm {H}^0(X,\Omega _X^2)$ which induces isomorphisms

$$\begin{aligned} \bigwedge ^p{\mathcal {T}}_X \simeq \Omega ^p_X \end{aligned}$$

which, in turn, induce a graded ${\mathbb {C}}$-algebra isomorphism

4.1 $$\begin{aligned} \mathrm {HT}^*(X)= \mathrm {H}^*\left( X,\bigwedge ^*{\mathcal {T}}_X\right) \simeq \mathrm {H}^*(X,\Omega ^*_X)\simeq \mathrm {H}^*(X,{\mathbb {C}}). \end{aligned}$$

Thus, as a graded ${\mathbb {C}}$-algebra, the ring of polyvectors is isomorphic to the de Rham cohomology.

In this note, we are mostly interested in another viewpoint of the polyvector fields. Namely, the ring of polyvectors acts on the de Rham cohomology by contraction. That is, given $v\in \mathrm {H}^q(X,\bigwedge ^p {\mathcal {T}}_X)$ and $x\in \mathrm {H}^{q'}(X,\Omega _X^{p'})$ the action is defined as

$$\begin{aligned} v \lrcorner x \in \mathrm {H}^{q+q'}(X,\Omega _X^{p'-p}). \end{aligned}$$

The following is immediate, see also [12, Lem. 2.4].

### Lemma 4.2

For *X* a hyper-Kähler manifold the de Rham cohomology is a free module of rank one over the polyvector fields generated by a Calabi–Yau form $\sigma ^n \in \mathrm {H}^0(X,\Omega _X^{2n})$.

The reason why the ring of polyvectors is of interest to us is the following crucial result. It relies on the modified Hochschild–Konstant–Rosenberg isomorphism identifying Hochschild (co)homology with polyvectors and the de Rham cohomology [4].

### Theorem 4.3

A derived equivalence $\Phi :\mathrm {D}^b(X)\xrightarrow {\sim } \mathrm {D}^b(Y)$ induces naturally a ${\mathbb {C}}$-algebra isomorphism $\Phi ^{\mathrm {HT}}:\mathrm {HT}^*(X)\xrightarrow {\sim } \mathrm {HT}^*(Y)$ such that the action of the polyvector fields is equivariant for the induced isomorphism $\Phi ^{\mathrm {H}}:\mathrm {H}^*(X,{\mathbb {C}})\xrightarrow {\sim } \mathrm {H}^*(Y,{\mathbb {C}})$.

Spelling this out, for $v\in \mathrm {HT}^*(X)$ and $x\in \mathrm {H}^*(X,{\mathbb {C}})$ we have

$$\begin{aligned} \Phi ^{\mathrm {H}}(v\lrcorner x)=\Phi ^{\mathrm {HT}}(v)\lrcorner \Phi ^{\mathrm {H}}(x)\in \mathrm {H}^*(Y,{\mathbb {C}}). \end{aligned}$$

## Reinventing the LLV Lie Algebra

We will define a new Lie algebra, which will turn out to be isomorphic to ${\mathfrak {g}}(X)$ with scalars extended to ${\mathbb {C}}$. This will prove Theorem 1.1 from the introduction.

Recall that *X* is a hyper-Kähler manifold of dimension 2*n*. We consider the holomorphic grading operator $h_p$ and the antihomolorphic grading operator $h_q$ defined by acting on $\mathrm {H}^{k,l}(X)$ via

$$\begin{aligned} h_p=(k-n) \mathrm {id}, \quad h_q = (l-n) \mathrm {id}. \end{aligned}$$

To avoid confusions, the indices *p* and *q* do not relate to *k* or *l* in any way, but just refer to the standard convention that the holomorphic degree of a smooth form is usually denoted by *p* and the antiholomorphic degree of a form by *q*.

With these definitions the usual grading operator *h* for the cohomological grading is just $h=h_p+h_q$. We define the Hodge grading operator $h':=h_q-h_p$.With this definition the action of the polyvector fields $\mathrm {HT}^*(X)$ on the de Rham cohomology $\mathrm {H}^*(X,{\mathbb {C}})$ alluded to in Lemma 4.2 has degree two with respect to the grading $h'$.

For $\mu \in \mathrm {HT}^2(X)$ we define the operator

$$\begin{aligned} e_\mu :=\mu \lrcorner \_ \in End (\mathrm {H}^*(X,{\mathbb {C}})). \end{aligned}$$

We say that $\mu$ is Hard Lefschetz if the operator $e_\mu$ satisfies the Hard Lefschetz isomorphisms with respect to the grading operator $h'$. The Jacobson–Morozov theorem asserts that this is equivalent to the existence of an operator $f_\mu \in End (\mathrm {H}^*(X,{\mathbb {C}}))$ such that

$$\begin{aligned} (e_\mu ,h',f_\mu ) \end{aligned}$$

generates a Lie subalgebra of $End (\mathrm {H}^*(X,{\mathbb {C}}))$ isomorphic to $\mathfrak {sl}_2$.

### Definition 5.1

The complex Lie algebra ${\mathfrak {g}}'(X)$ is defined to be the smallest Lie subalgebra of $End (\mathrm {H}^*(X,{\mathbb {C}}))$ containing all $\mathfrak {sl}_2$-triples $(e_\mu ,h',f_\mu )$ for all Hard Lefschetz $\mu \in \mathrm {HT}^2(X)$.

Equivalently, one could have defined the Lie algebra ${\mathfrak {g}}'(X)$ as the Lie subalgebra of the endomorphism algebra $End (\mathrm {HT}^*(X))$ containing all $\mathfrak {sl}_2$-triples with $\mu$ Hard Lefschetz. Through the isomorphism

$$\begin{aligned} \mathrm {HT}^*(X)\lrcorner \sigma ^n \simeq \mathrm {H}^*(X,{\mathbb {C}}) \end{aligned}$$

these two definitions are identified.

Recall from (4.1) that the choice of a symplectic form produces an abstract graded ${\mathbb {C}}$-algebra isomorphism

$$\begin{aligned} \mathrm {HT}^*(X)\simeq \mathrm {H}^*(X,\Omega _X^*)\simeq \mathrm {H}^*(X,{\mathbb {C}}). \end{aligned}$$

Thus, the choice of a symplectic form leads to the following result.

### Lemma 5.2

There is an isomorphism of complex Lie algebras

$$\begin{aligned} {\mathfrak {g}}(X)\otimes _{{\mathbb {Q}}} {\mathbb {C}} \simeq {\mathfrak {g}}'(X). \end{aligned}$$

We also deduce the following consequence from Theorem 4.3.

### Proposition 5.3

For a derived equivalence between hyper-Kähler manifolds $\Phi :\mathrm {D}^b(X)\simeq \mathrm {D}^b(Y)$ the isomorphism

$$\begin{aligned} \Phi ^{\mathrm {HT}}:\mathrm {HT}^2(X)\xrightarrow {\sim } \mathrm {HT}^2(Y) \end{aligned}$$

induces naturally a Lie algebra isomorphism

$$\begin{aligned} \Phi ^{{\mathfrak {g}}}:{\mathfrak {g}}'(X)\xrightarrow {\sim } {\mathfrak {g}}'(Y) \end{aligned}$$

such that the induced isomorphism

$$\begin{aligned} \Phi ^{\mathrm {H}}:\mathrm {H}^*(X,{\mathbb {C}})\xrightarrow {\sim } \mathrm {H}^*(Y,{\mathbb {C}}) \end{aligned}$$

is equivariant with respect to $\Phi ^{{\mathfrak {g}}}$.

Spelling this again out means that for $j\in {\mathfrak {g}}'(X)$ and $x\in \mathrm {H}^*(X,{\mathbb {C}})$ we have

$$\begin{aligned} \Phi ^{\mathrm {H}}(j.x)=\Phi ^{{\mathfrak {g}}}(j).\Phi ^{\mathrm {H}}(x)\in \mathrm {H}^*(Y,{\mathbb {C}}). \end{aligned}$$

The connection between all that has been said so far and the main tool for all the applications we will present is the following main theorem of [12] which was also implicitely proven (but not stated in the form below) by Verbitsky [14].

### Theorem 5.4

The Lie algebras ${\mathfrak {g}}(X)\otimes _{{\mathbb {Q}}} {\mathbb {C}}$ and ${\mathfrak {g}}'(X)$ are equal as Lie subalgebras of the Lie algebra $End (\mathrm {H}^*(X,{\mathbb {C}}))$.

### Proof

Verbitsky showed that there is an isomorphism of ungraded vector spaces

$$\begin{aligned} \eta :\mathrm {H}^{*}(X,{\mathbb {C}})\xrightarrow {\sim } \mathrm {H}^{*}(X,{\mathbb {C}}). \end{aligned}$$

The explicit description of $\eta$ is not import, we only need the following two properties shwon by Verbitsky. Firstly, $\eta$ conjugates the two Lie algebras, i.e.

$$\begin{aligned} \eta \left( {\mathfrak {g}}(X)\otimes _{{\mathbb {Q}}} {\mathbb {C}} \right) \eta ^{-1}={\mathfrak {g}}'(X). \end{aligned}$$

Secondly, the isomorphism $\eta$ is obtained by integrating the action of the Lie algebra ${\mathfrak {g}}(X)$, that is it lies in the subgroup of automorphism $\mathrm {Aut}(\mathrm {H}^*(X,(C)))$ generated by integrated operators of ${\mathfrak {g}} \otimes _{{\mathbb {Q}}} {\mathbb {C}}$. Since all such operators $\mu$ contained in the above subgroup satisfy

$$\begin{aligned} \mu \left( {\mathfrak {g}}(X)\otimes _{{\mathbb {Q}}} {\mathbb {C}} \right) \mu ^{-1}={\mathfrak {g}}(X) \otimes _{{\mathbb {Q}}} {\mathbb {C}}. \end{aligned}$$

one can conclude the proof.

We will, however, follow Taelman’s proof. From Lemma 5.2 we infer that it is enough to show only the inclusion

$$\begin{aligned} {\mathfrak {g}}'(X)\subset {\mathfrak {g}}(X) \otimes _{\mathbb {Q}} {\mathbb {C}}. \end{aligned}$$

A straightforward calculation shows that

$$\begin{aligned} (e_\sigma ,h_p,e_{{\check{\sigma }}}) \end{aligned}$$

is an $\mathfrak {sl}_2$-triple, where ${\check{\sigma }}\in \mathrm {H}^0(\bigwedge ^2({\mathcal {T}}_X))$ is the dual symplectic form (note that the Lefschetz operator $e_\sigma$ acts via cup product, whereas $e_{{\check{\sigma }}}$ acts by contraction of polyvector fields).

Analogously or using Hodge symmetry, for the complex conjugate form ${\bar{\sigma }}\in \mathrm {H}^2(X,{\mathcal {O}}_X)$ the operator $e_{{\bar{\sigma }}}$ has the Hard Lefschetz property for the grading operator $h_q$. The Jacobson–Morozov Theorem grants the existence of an operator $g\in End (\mathrm {H}^*(X,{\mathbb {C}}))$ such that

$$\begin{aligned} (e_{{\bar{\sigma }}},h_q,g) \end{aligned}$$

forms an $\mathfrak {sl}_2$-triple. An easy check shows that all elements from the $\mathfrak {sl}_2$-triple $(e_\sigma ,h_p,e_{{\check{\sigma }}})$ commute with all elements from the $\mathfrak {sl}_2$-triple $(e_{{\bar{\sigma }}},h_q,g)$. For example, $e_\sigma$ and $e_{{\bar{\sigma }}}$ commute as the de Rham cohomology is graded-commutative and the operators $e_\sigma$ and $e_{{\check{\sigma }}}$ commute with $h_q$, because they does not change the antiholomorphic degree of a form. Similar arguments apply to the other operators. Thus we obtain two new $\mathfrak {sl}_2$-triples

$$\begin{aligned} (e_\sigma +e_{{\bar{\sigma }}},h,e_{{\check{\sigma }}}+g), \quad (e_\sigma -e_{{\bar{\sigma }}},h,e_{{\check{\sigma }}}-g). \end{aligned}$$

This gives that $e_{{\check{\sigma }}}\in {\mathfrak {g}}(X)\otimes _{\mathbb {Q}} {\mathbb {C}}$. Since $[e_\sigma ,e_{{\check{\sigma }}}]=h_p$ and $h_p+h_q=h$, we deduce furthermore that $h_p, h_q$ and therefore $h'=h_q-h_p$ are all contained inside ${\mathfrak {g}}(X)\otimes _{\mathbb {Q}} {\mathbb {C}}$.

Since evidently $e_{{\bar{\sigma }}}$ is also contained in ${\mathfrak {g}}(X)\otimes _{\mathbb {Q}} {\mathbb {C}}$ (the action via contraction of polyvector fields agrees with the cup product), it is left to show that for almost all $\mu \in \mathrm {H}^1(X,{\mathcal {T}}_X)$ the operator $e_\mu$ lies in ${\mathfrak {g}}(X)\otimes _{\mathbb {Q}} {\mathbb {C}}$. This follows from the identity

$$\begin{aligned} {[}e_{{\check{\sigma }}},e_\eta ]=e_\mu \end{aligned}$$

for $$\eta \in \mathrm {H}^1(X,\Omega _X)$$ satisfying

$$\begin{aligned} \mu ={\check{\sigma }}\lrcorner \eta \in \mathrm {H}^1(X,{\mathcal {T}}_X) \end{aligned}$$

which follows from a straightforward calculation, see [12, Lem. 2.13]. $\square$

The theorem implies that the isomorphism $\Phi ^{{\mathfrak {g}}}$ from Proposition 5.3 is already defined over ${\mathbb {Q}}$, since the same holds for the induced isomorphism on singular cohomology. We thus have proved Theorem 1.1 which we state her again for the reader’s convenience.

### Corollary 5.5

A derived equivalence $\Phi :\mathrm {D}^b(X)\xrightarrow {\sim } \mathrm {D}^b(Y)$ between hyper-Kähler manifolds induces naturally a Lie algebra isomorphism

$$\begin{aligned} \Phi ^{{\mathfrak {g}}}:{\mathfrak {g}}(X)\xrightarrow {\sim } {\mathfrak {g}}(Y) \end{aligned}$$

such that the induced isomorphism

$$\begin{aligned} \Phi ^{\mathrm {H}}:\mathrm {H}^*(X,{\mathbb {Q}})\xrightarrow {\sim } \mathrm {H}^*(Y,{\mathbb {Q}}) \end{aligned}$$

is equivariant with respect to $\Phi ^{{\mathfrak {g}}}$.

## Verbitsky Component and Extended Mukai Lattice

We want to draw consequences of Theorem 5.4 for the study of derived equivalences of hyper-Kähler manifolds and their induced actions on cohomology.

### Definition 6.1

The Verbitsky component $\mathrm {SH}(X,{\mathbb {Q}})\subset \mathrm {H}^*(X,{\mathbb {Q}})$ is the subalgebra generated by $\mathrm {H}^2(X,{\mathbb {Q}})$.

It is easy to see that the Verbitsky component is an irreducible representation of the LLV Lie algebra ${\mathfrak {g}}(X)$ and it is characterized as such as the irreducible representation whose Hodge structure attains the maximal possible width. It is equipped with the Mukai pairing $b_{\mathrm {SH}}$ defined via

$$\begin{aligned} b_{\mathrm {SH}}(\lambda _1\dots \lambda _m,\mu _1\cdots \mu _{2n-m}):=(-1)^m \int _X \lambda _1\cdots \lambda _m \mu _1\cdots \mu _{2n-m} \end{aligned}$$

for classes $\lambda _i,\mu _j\in \mathrm {H}^2(X,{\mathbb {Q}})$ which agrees with the generalized Mukai pairing alluded to in Remark 2.2.

### Corollary 6.2

For a derived equivalence $\Phi :\mathrm {D}^b(X)\xrightarrow {\sim } \mathrm {D}^b(Y)$ between hyper-Kähler manifolds the induced isomorphism $\Phi ^{\mathrm {H}}$ restricts to a Hodge isometry

$$\begin{aligned} \Phi ^{\mathrm {SH}}:\mathrm {SH}(X,{\mathbb {Q}})\xrightarrow {\sim } \mathrm {SH}(Y,{\mathbb {Q}}). \end{aligned}$$

### Proof

Since the Verbitsky component is the unique irreducible representation whose Hodge strucutre attains the maximal possible width and by Theorem 1.1 the isomorphism $\Phi ^{\mathrm {H}}$ respects the LLV algebra, we conclude that $\Phi ^{\mathrm {H}}$ must restrict to an isomorphism of the Verbitsky component. The Mukai pairing on the Verbitsky component agrees with the generalized Mukai pairing, which is a derived invariant. $\square$

We want to study the Verbitsky component and the LLV Lie algebra more closely to further refine the study of $Aut (\mathrm {D}^b(X))$.

### Definition 6.3

The rational quadratic vector space defined by

$$\begin{aligned} \tilde{H }(X,{\mathbb {Q}}) :={\mathbb {Q}} \alpha \oplus \mathrm {H}^2(X,{\mathbb {Q}}) \oplus {\mathbb {Q}} \beta . \end{aligned}$$

is called the extended Mukai lattice. Its quadratic form ${\tilde{b}}$ restricts to the Beauville–Bogomolov–Fujiki form *b* on $\mathrm {H}^2(X,{\mathbb {Q}})$ [6, Sec. 23] and the two classes $\alpha$ and $\beta$ are orthogonal to $\mathrm {H}^2(X,{\mathbb {Q}})$ and satisfy ${\tilde{b}}(\alpha ,\beta )=-1$ as well as ${\tilde{b}}(\alpha , \alpha )= {\tilde{b}}(\beta ,\beta )=0$.

Furthermore, we define on $\tilde{\mathrm {H}}(X,{\mathbb {Q}})$ a grading by declaring $\alpha$ to be of degree $-2$, $\mathrm {H}^2(X,{\mathbb {Q}})$ sits in degree zero and $\beta$ is of degree two. Finally, the extended Mukai lattice is equipped with a weight-two Hodge structure

$$\begin{aligned} (\tilde{\mathrm {H}}(X,{\mathbb {Q}})\otimes {\mathbb {C}})^{2,0}&:=\mathrm {H}^{2,0}(X)\\ (\tilde{\mathrm {H}}(X,{\mathbb {Q}})\otimes {\mathbb {C}})^{0,2}&:=\mathrm {H}^{0,2}(X)\\ (\tilde{\mathrm {H}}(X,{\mathbb {Q}})\otimes {\mathbb {C}})^{1,1}&:=\mathrm {H}^{1,1}(X) \oplus {\mathbb {C}} \alpha \oplus {\mathbb {C}} \beta . \end{aligned}$$

There exists a graded morphism  sitting in the following short exact sequenceHere, the map $\Delta _n$ is the Laplacian operator defined on pure tensors viaSurjectivity follows easily from the fact that the symmetric power $\mathrm {Sym}^kV$ of a vector space *V* is generated by $v\cdots v$ for all $v\in V$. The map $\psi$ is uniquely determined (up to scaling) by the condition that it is a morphism of ${\mathfrak {g}}(X)$-modules. The ${\mathfrak {g}}(X)$-structure of $\tilde{\mathrm {H}}(X,{\mathbb {Q}})$ is defined by $e_\omega (\alpha )=\omega$, $e_\omega (\mu )=b(\omega ,\mu )\beta$ and $e_\omega (\beta )=0$ for all classes $\omega , \mu \in \mathrm {H}^2(X,{\mathbb {Q}})$. The *n*-th symmetric power $\mathrm {Sym}^n(\tilde{\mathrm {H}}(X,{\mathbb {Q}}))$ then inherits the structure of a ${\mathfrak {g}}(X)$-module by letting ${\mathfrak {g}}(X)$ act by derivations. We fix once and for all a choice of $\psi$ by setting $\psi (1)=\alpha ^n/n!$. By Schur’s lemma, $\psi$ is injective.

Taelman [12, Sec. 3] showed that the map $\psi$ is an isometry with respect to the Mukai pairing on $SH (X,{\mathbb {Q}})$ and the pairing

$$\begin{aligned} b_{[n]}(x_1 \cdots x_n, y_1 \cdots y_n)=(-1)^n c_X \sum _{\sigma \in {\mathfrak {S}}_n}\prod _{i=1}^n {\tilde{b}}(x_i,y_{\sigma (i)}) \end{aligned}$$

on $\mathrm {Sym}^n(\tilde{\mathrm {H}}(X,{\mathbb {Q}}))$, where $c_X$ is the Fujiki constant characterized by the property

$$\begin{aligned} \int _X\omega ^{2n}=c_X\frac{(2n)!}{2^nn!}b(\omega ,\omega )^n \end{aligned}$$

for all $\omega \in \mathrm {H}^2(X,{\mathbb {Q}})$. Note that our definition of $b_{[n]}$ differs from Taelman’s definition by the Fujiki constant. Ours has the advantage that $\psi$ is always an isometry.

Summing up, the inclusion $\psi$ respects the

- $${\mathfrak {g}}(X)$$-module structure,
- quadratic forms,
- Hodge structures,
- gradings.

## Action of Derived Equivalences on the Extended Mukai Lattice

Recall that we have deduced the existence of a representation7.1and the isometries in the image of this representation normalize the action of the LLV algebra ${\mathfrak {g}}(X)$, i.e. for these $g\in \mathrm {O}(\mathrm {SH}(X,{\mathbb {Q}}))$ we have

$$\begin{aligned} g {\mathfrak {g}}(X)g^{-1}={\mathfrak {g}}(X) \subset End (\mathrm {SH}(X,{\mathbb {Q}})). \end{aligned}$$

Let us study these automorphisms a bit further.

### Definition 7.1

The group $Aut (\mathrm {SH}(X,{\mathbb {Q}}),b_{\mathrm {SH}},{\mathfrak {g}}(X))$ is the group of all isometries of the Verbitsky component that normalize the action of the LLV algebra.

The main representation-theoretic input for our discussion is the following result [12, Sec. 4].

### Proposition 7.2

If *n* is odd or the second Betti number is odd, then

$$\begin{aligned} Aut (\mathrm {SH}(X,{\mathbb {Q}}),b_{\mathrm {SH}},{\mathfrak {g}}(X)) \simeq \mathrm {O}(\tilde{\mathrm {H}}(X,{\mathbb {Q}})). \end{aligned}$$

We make this isomorphism more explicit. Let *X* and *Y* be deformation-equivalent hyper-Kähler manifolds together with a derived equivalence $\Phi :\mathrm {D}^b(X) \xrightarrow {\sim } \mathrm {D}^b(Y)$. Then there exists a unique Hodge isometry

$$\begin{aligned} \Phi ^{\tilde{\mathrm {H}}}:\tilde{\mathrm {H}}(X,{\mathbb {Q}}) \xrightarrow {\sim } \tilde{\mathrm {H}}(Y,{\mathbb {Q}}) \end{aligned}$$

inducing the following commutative diagram 
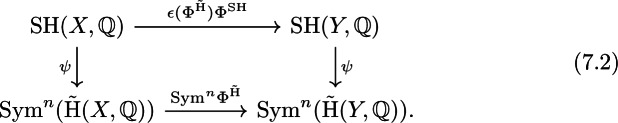


The scalar $\epsilon (\Phi ^{\tilde{\mathrm {H}}})\in \{\pm 1\}$ depends on defining orientations on the vector spaces $\tilde{\mathrm {H}}(X,{\mathbb {Q}})$ respectively $\tilde{\mathrm {H}}(Y,{\mathbb {Q}})$ and for $X=Y$ we simply have $\epsilon (\Phi ^{\tilde{\mathrm {H}}}) = \det (\Phi ^{\tilde{\mathrm {H}}})^{n+1}$. In particular, in the case $X=Y$, the representation (7.1) factors via the commutative diagram 
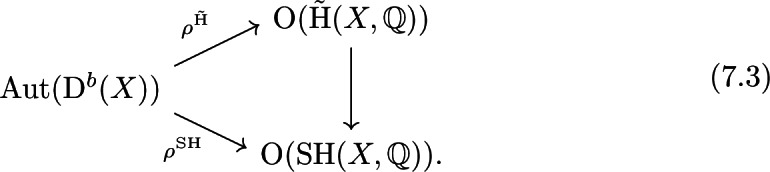


### Remark 7.3

In all known examples, derived equivalent hyper-Kähler manifolds are deformation-equivalent, but this is not known in general. Without this assumption, the above proposition has to be weakened as we shall demonstrate.

One can, using similitudes, still formulate a version of Proposition 7.2 in the general case. This will be needed in the next section for the application to Hodge structures.

### Theorem 7.4

Let *X* and *Y* be arbitrary hyper-Kähler manifolds and $\Phi :\mathrm {D}^b(X)\xrightarrow {\sim } \mathrm {D}^b(Y)$ be a derived equivalence. Then there exists a Hodge similitude  and a scalar $\lambda \in {\mathbb {Q}}^*$ such that 
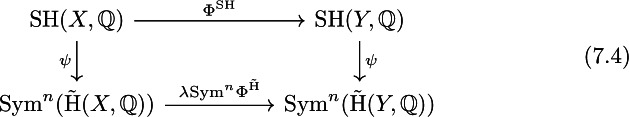
 commutes.

## Hodge Structures

In this section we want to give one application of the results presented so far regarding derived equivalent hyper-Kähler manifolds and their Hodge structures. We first want to recall a recent result of Soldatenkov [11],^1^ whose statement and proof are similar in flavour to what we will discuss afterwards for derived equivalences.

### Theorem 8.1

Let *X* and *Y* be arbitrary hyper-Kähler manifolds and $\varphi :\mathrm {H}^2(X,{\mathbb {Q}})\xrightarrow {\sim } \mathrm {H}^2(Y,{\mathbb {Q}})$ be an isomorphism of ${\mathbb {Q}}$-Hodge structures, which is the restriction of a global algebra automorphism $\phi :\mathrm {H}^*(X,{\mathbb {Q}})\xrightarrow {\sim } \mathrm {H}^*(Y,{\mathbb {Q}})$. Then for all $i\in {\mathbb {Z}}$ the restrictions

$$\begin{aligned} \phi :\mathrm {H}^i(X,{\mathbb {Q}})\xrightarrow {\sim } \mathrm {H}^i(Y,{\mathbb {Q}}) \end{aligned}$$

are isomorphisms of ${\mathbb {Q}}$-Hodge structures.

### Proof

We briefly sketch the argument. Since $\phi$ is a graded algebra automorphism, the adjoint action produces an isomorphism

$$\begin{aligned} \mathrm {ad}(\phi ) :{\mathfrak {g}}(X) \xrightarrow {\sim } {\mathfrak {g}}(Y). \end{aligned}$$

The fact that $\phi$ is graded implies that $\mathrm {ad}(\phi )(h)=h$. Moreover, the restriction of $\phi$ to $\mathrm {H}^2(X,{\mathbb {Q}})$ respects the Hodge structures. This implies that $\mathrm {ad}(\phi )(h')=h'$, where again $h'= h_q-h_p$. Indeed, the adjoint action of $\phi$ is determined by its restriction to the degree two component [11, Prop. 2.11]. As the morphism $\phi$ respects the Hodge structure on the second cohomology, the claim follows.

Since $h+h'=2h_q$ and $h-h'=2h_p$ we deduce $\mathrm {ad}(\phi )(h_p)=h_p$ and $\mathrm {ad}(\phi )(h_q)=h_q$. This is equivalent to $\phi$ being a morphism of ${\mathbb {Q}}$-Hodge structures. $\square$

The assertion that the isomorphism of Hodge structures is the restriction of a global algebra automorphism is frequently met. For example, Hodge isometries with positive determinant can be extended to algebra automorphisms of the even cohomology by integrating the LLV action. For more details and examples we refer to [11].

With this in mind, we can now prove the following result of Taelman [12, Sec. 5]. It also establishes a conjecture of Orlov in the case of hyper-Kähler manifolds [10] stating that derived equivalent varieties have the same Hodge numbers.

### Theorem 8.2

Let *X* and *Y* be derived equivalent hyper-Kähler manifolds. Then for all $i\in {\mathbb {Z}}$ we have an isomorphism

$$\begin{aligned} \mathrm {H}^i(X,{\mathbb {Q}})\simeq \mathrm {H}^i(Y,{\mathbb {Q}}) \end{aligned}$$

of ${\mathbb {Q}}$-Hodge structures.

### Proof

Let us denote by $\Phi$ a derived equivalence between *X* and *Y*. Recall from [8, 13] the Lie algebra isomorphism ${\mathfrak {g}}(X)\simeq \mathfrak {so}(\tilde{\mathrm {H}}(X,{\mathbb {Q}}))$ (in loc. cit. the isomorphism is only stated over ${\mathbb {R}}$. For the statement with rational coefficients, see [11, Prop. 2.9].). Composing this isomorphism with $\Phi ^{{\mathfrak {g}}}$ we obtain a Lie algebra isomorphism

$$\begin{aligned} \mathfrak {so}(\tilde{\mathrm {H}}(X,{\mathbb {Q}})) \simeq \mathfrak {so}(\tilde{\mathrm {H}}(Y,{\mathbb {Q}})). \end{aligned}$$

Every such Lie algebra isomorphism is equal to $\mathrm {ad}(\phi )$ for some , see [12, Prop. 4.1] which is the analogue of Proposition 7.2 in this case. Theorem 7.4 now implies that $\phi$ must be a Hodge similitude. More precisely, it differs from $\Phi ^{\tilde{\mathrm {H}}}$ only by a scalar.

Using

$$\begin{aligned} \tilde{\mathrm {H}}(X,{\mathbb {Q}}) \simeq {\mathbb {Q}}\alpha \oplus {\mathbb {Q}}\beta \oplus \mathrm {NS}(X)_{{\mathbb {Q}}} \oplus \mathrm {T}(X)_{{\mathbb {Q}}} \end{aligned}$$

and Witt cancelation for quadratic spaces, one easily shows that there exists a Hodge isometry $\gamma \in \mathrm {SO}(\tilde{\mathrm {H}}(Y,{\mathbb {Q}}))$ such that the composition $\gamma \circ \phi$ is now a graded Hodge similitude, i.e. $\alpha$ and $\beta$ are mapped to multiples of themselves. By definition, this implies that the adjoint morphism of $\gamma \circ \phi$ satisfies

8.1 $$\begin{aligned} \mathrm {ad}(\gamma \circ \phi )(h)=h, \quad \mathrm {ad}(\gamma \circ \phi )(h')=h'. \end{aligned}$$

Let us for the moment assume that we can find a global algebra isomorphism $\eta :\mathrm {H}^*(Y,{\mathbb {Q}})\xrightarrow {\sim } \mathrm {H}^*(Y,{\mathbb {Q}})$ whose adjoint action equals $\gamma$ as isomorphisms of the LLV Lie algebra ${\mathfrak {g}}(Y)$. Then we can consider the composition

$$\begin{aligned} \eta \circ \Phi ^{\mathrm {H}}:\mathrm {H}^*(X,{\mathbb {Q}})\xrightarrow {\sim } \mathrm {H}^*(Y,{\mathbb {Q}}). \end{aligned}$$

From (8.1) we infer again that $\mathrm {ad}(\eta \circ \Phi ^{\mathrm {H}})(h)=h$ and $\mathrm {ad}(\eta \circ \Phi ^{\mathrm {H}})(h')=h'$. As in the proof of Theorem 8.1 this implies that $\eta \circ \Phi ^{\mathrm {H}}$ induces in each degree the desired isomorphism of Hodge structures.

It is left to prove the existence of the global algebra isomorphism $\eta$. In general, integrating the action of the LLV algebra ${\mathfrak {g}}(X)$ produces an action of $\mathrm {SO}(\tilde{\mathrm {H}}(Y,{\mathbb {Q}}))$ on the even cohomology $\mathrm {H}^{2*}(Y,{\mathbb {Q}})$ [11, Prop. 2.10]. To construct an algebra automorphism of the full cohomology $\mathrm {H}^{*}(Y,{\mathbb {Q}})$ one uses the ${\mathbb {Q}}$-algebraic group $\mathrm {GSpin}$. More precisely, one uses the natural surjectionto lift $\gamma$ and constructs an action of $\mathrm {GSpin}(\tilde{\mathrm {H}}(Y,{\mathbb {Q}}))$ on the full cohomology such that the induced action of $\mathrm {Spin}(\tilde{\mathrm {H}}(Y,{\mathbb {Q}})) \subset \mathrm {GSpin}(\tilde{\mathrm {H}}(Y,{\mathbb {Q}}))$ is the integrated action of the LLV algebra. For details we refer to [12, Sec. 5]. $\square$

## Further Results

We have presented the first six sections of [12]. In the remaining part of loc. cit. the representation $\rho ^{\tilde{\mathrm {H}}}$ from (7.3) is further studied. The main result is a bound on the image of $\rho ^{\tilde{\mathrm {H}}}$ in terms of (subgroups) of the orthogonal group $\mathrm {O}(\Lambda )$ some lattice

$$\begin{aligned} \Lambda \subset \tilde{\mathrm {H}}(X,{\mathbb {Q}}) \end{aligned}$$

for *X* (a deformation of) the Hilbert scheme of two points on a K3 surface.

In [1], building upon the results presented so far, the study of derived categories of projective hyper-Kähler manifolds is further refined. The main technical tool is a Mukai vector taking values in the extended Mukai lattice $\tilde{\mathrm {H}}(X,{\mathbb {Q}})$. This yields structural results for derived categories and derived equivalences for general hyper-Kähler varieties as well as many generalisations of results known for derived categories of K3 surfaces to the case of higher-dimensional deformations of Hilbert schemes.

## Footnotes

1 We thank Andrey Soldatenkov for a stimulating conversation about his results.
